# The functions and potential roles of extracellular vesicle noncoding RNAs in gynecological malignancies

**DOI:** 10.1038/s41420-021-00645-3

**Published:** 2021-09-22

**Authors:** Lulu Si, Jing Bai, Hanlin Fu, Haifeng Qiu, Ruixia Guo

**Affiliations:** 1 Department of Gynecology, The First Affiliated Hospital of Zhengzhou University, Zhengzhou, China; 2 Medical Key Laboratory for Prevention and Treatment of Malignant Gynecological Tumor, Zhengzhou, Henan China

**Keywords:** Gynaecological cancer, Oncogenesis

## Abstract

Extracellular vesicles (EVs) are small membranous vesicles secreted by multiple kinds of cells and are widely present in human body fluids. EVs containing various constituents can transfer functional molecules from donor cells to recipient cells, thereby mediating intercellular communication. Noncoding RNAs (ncRNAs) are a type of RNA transcript with limited protein-coding capacity, that have been confirmed to be enriched in EVs in recent years. EV ncRNAs have become a hot topic because of their crucial regulating effect in disease progression, especially in cancer development. In this review, we summarized the biological functions of EV ncRNAs in the occurrence and progression of gynecological malignancies. In addition, we reviewed their potential applications in the diagnosis and treatment of gynecological malignancies.

## Facts

- Extracellular vesicles are small membranous vesicles containing various constituents.
- Noncoding RNAs are functional transcripts with limited protein-coding capacity.
- Extracellular vesicle noncoding RNAs regulate the occurrence and progression of gynecological malignancies.
- Extracellular vesicle noncoding RNAs may be applied in the diagnosis and treatment of gynecological malignancies

## Open questions

- What are the functions of extracellular vesicle noncoding RNAs on the development of gynecological malignancies?
- How are extracellular vesicle noncoding RNAs involved in the progression of gynecological malignancies?
- How can we employ extracellular vesicle noncoding RNAs to monitor and treat gynecological malignancies?

## Introduction

Gynecological malignancies, mainly cervical cancer (CC), ovarian cancer (OC) and endometrial cancer (EC), are a worldwide problem that seriously endanger women’s health and lives. Although progress has been made in the prevention, screening, diagnosis and treatment of these cancers over the past decades, many issues remain to be solved, such as the early diagnosis and chemoresistance of OC [1] and the recurrence and metastasis of CC [2] and EC [3].

Noncoding RNAs (ncRNAs), once considered to be transcriptional noise for not coding proteins, are classified into different categories: microRNAs (miRNAs), long noncoding RNAs (lncRNAs), circular RNAs (circRNAs) and other types [4]. Recently increasing evidence has shown that ncRNAs play a vital role in various physiological and pathophysiological processes via transcriptional and posttranscriptional regulation. It has been reported that aberrant expression of miRNAs, lncRNAs and circRNAs is associated with cancer progression [5, 6]. Notably, ncRNAs employ extracellular vesicles (EVs) to reach recipient cells or tissues, acting as messengers of intercellular communication [7–9]. EVs are small membranous vesicles with a heterogeneous bilayer phospholipid structure [10] that are produced by multiple types of cells and identified in various body fluids, such as serum, plasma, urine, saliva, ascites, pleural fluid, and breast milk. EVs contain abundant bioactive components, including proteins, lipids, DNA, mRNA, ncRNAs and metabolites [11, 12], and mediate intercellular communication by transferring these molecules from donor cells to recipient cells to perform numerous functions [13]. Accumulating evidence indicates that EV ncRNAs are widely involved in cancer occurrence, progression, metastasis and chemoresistance [7, 8, 14]. In this review, we summarized the current studies about EV ncRNAs and their emerging roles in affecting the processes of gynecological malignancies. EV miRNAs, lncRNAs and circRNAs were discussed, highlighting the major contribution of EV miRNAs and lncRNAs to CC and OC. In addition, we investigated their potential applications in the diagnosis and treatment of gynecological malignancies, providing new insights into the development of biomarkers and anticancer therapy.

## The functional roles of ncRNAs in gynecological malignancies

ncRNAs are functional transcripts with limited protein-coding capacity, that can be divided into different classes, mainly according to their length. Here, we primarily focused on miRNAs, lncRNAs and circRNAs and briefly discussed their roles in regulating gynecological malignancy progression.

miRNAs are short ncRNAs of ~22 nucleotides (nt) in length that regulate the expression of mRNAs. miRNAs degrade mRNA or repress their translation by binding to their 3’ untranslated regions (3’ UTRs) with complementary sequences [15]. Ultimately, miRNAs seem to function as oncogenes or tumor suppressors in cancer, including gynecological malignancies. For instance, MIR-G-1, acting as an oncogene in CC, can activate the WNT-CTNNB1/β-catenin signaling pathway by interacting with WNT7B, thereby upregulating TMED5 to promote cell proliferation, migration, invasion and tumor growth [16]. These oncogenic miRNAs have the potential to serve as targets in anticancer therapy. However, in OC, miR-450a reduced tumor migration, invasion and growth in vivo and in vitro, by modulating energetic metabolism and epithelial-mesenchymal transition (EMT), suggesting that it functions as a tumor suppressor [17]. Many studies suggest that these suppressive miRNAs are downregulated in cancers, such as CC, OC and EC.

ncRNAs greater than 200 nt in length are defined as lncRNAs. Unlike miRNAs, lncRNAs function in multifarious ways. It was reported that lncRNAs can not only bind to proteins but can also interact with mRNAs, miRNAs and DNA, exhibiting diverse mechanisms. However, it was reported that most lncRNAs in gynecological malignancies function by interacting with proteins or miRNAs. A multitude of lncRNAs, such as HOTAIR, H19, MALAT1, MEG3, LET and PVT1, are correlated with cancer development. Zhang et al. reported that MEG3 can directly bind to the P-STAT3 protein and promote the degradation of P-STAT3 via ubiquitination [18]. The instability of P-STAT3 further inhibits the proliferation and promotes the apoptosis of CC cells [18]. In addition, lncRNAs often act as sponges or competitive endogenous RNAs (ceRNAs) for miRNAs to prevent gene repression mediated by miRNA. For example, it was reported that HOTAIR, acting as an oncogenic molecular, is upregulated in CC. Li et al. reported that HOTAIR can competitively bind to miR-23b and regulate the expression of MAPK1, promoting the proliferation and invasion of CC cells [19]. Furthermore, lncRNAs can also act as scaffolds. Like lnc-CCDST in CC, lnc-CCDST can promote DHX9 degradation by providing a scaffold for the formation of DHX9 and MDM2 complexes. DHX9 degradation inhibits the migration and invasion of cancer cells [20]. Above all, lncRNAs can function as oncogenes or tumor suppressors via various regulatory mechanisms.

circRNAs are a special type of lncRNA with a single-stranded covalently closed RNA structure. Several circRNAs were aberrantly expressed in gynecological malignancies. It seems that circRNAs function similarly to lncRNA by serving as sponges of miRNA and scaffolds for proteins and regulating transcription and splicing. However, it was reported that most circRNAs in gynecological malignancies regulate protein expression by sponging miRNAs, as observed with circ101996 [21], circATP8A2 [22], circ0007534 [23] and circ0075341 [24] in CC and circEPSTI1 [25], circ0061140 [26], circWHSC1 [27] and circCELSR1 [28] in OC. Similarly, circRNAs can also promote or inhibit cancer development via these diverse mechanisms.

Although the three kinds of ncRNAs mentioned above were initially regarded as lacking protein-coding abilities, an increasing number of studies have revealed that some of them carrying small open reading frames can be translated into peptides or proteins [29]. The lncRNA HOXB-AS can be translated into a conserved 53-aa peptide, which inhibits colon cancer growth by suppressing glucose metabolism [30]. It has been reported that LINC-PINT [31], circRNA-SHPRH [32], FBXW7 [33] and AKT3 [34] can encode small peptides that suppresses tumorigenesis in certain cancers. In contrast, small peptides encoded by circPPP1R12A [35], LINC00998 [36] and LOC90024 [37] can promote cancer development. In addition, Kang et al. identified a small peptide, miPEP133, encoded by pri-miRNA miR-34a, which was highly expressed in the ovary and other tissues. Additionally, miPEP133 inhibited the migration and invasion of cancer cells [38]. These results suggest that small peptides encoded by ncRNAs are correlated with cancer progression. More functional small peptides are being discovered in gynecological malignancies.

## The biogenesis and characteristics of EVs

**Fig. 1:**
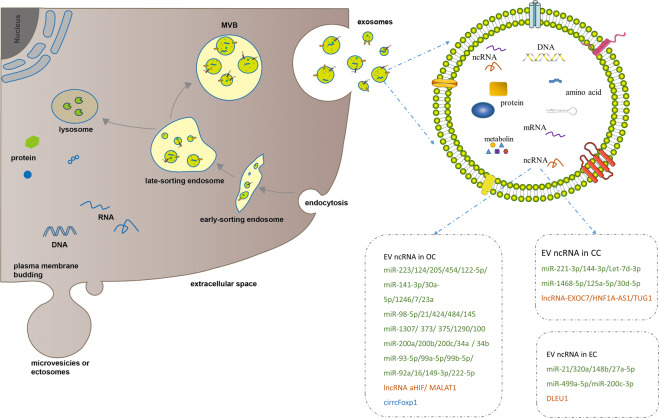
Biogenesis and contents of extracellular vesicles. Microvesicles (MVs) or ectosomes are released by direct budding from the plasma membrane. Exosomes are formed by inward invagination of the endosomal limiting membrane, and released after late-sorting endosomes (LSEs) fusion with cell membrane. Exosome contains proteins, DNAs, mRNAs, ncRNAs (miRNAs, long noncoding RNAs and circRNAs) and metabolites. Previously reported EV ncRNAs in CC, OC and EC were listed in the boxes. miRNAs, long noncoding RNAs and circRNAs were respectively labeled in green, orange and blue.

EVs can be divided into three types, mainly exosomes, microvesicles (MVs, also called ectosomes), and apoptotic bodies, on the basis of their biogenesis or cellular origin [39]. In consideration that apoptotic bodies are derived from the membranes of apoptotic cells, we primarily focused on exosomes and microvesicles. MVs are membranous vesicles 50–1000 nm in diameter that can be immediately released by direct budding from the plasma membrane [39]. However, exosomes, which are ~30–150 nm in diameter, have drawn increasing attention due to their unique characteristics. Exosomes are generated through a series of processes involving the formation of intracellular multivesicular bodies (MVBs) and intraluminal vesicles (ILVs) (Fig. 1). First, the invagination of the plasma membrane leads to the formation of early-sorting endosomes (ESEs). After maturing into late-sorting endosomes (LSEs), ESEs eventually generate MVBs containing ILVs by inward invagination of the endosomal limiting membrane. Last, ILVs are released into the extracellular milieu as exosomes upon the fusion of MVBs with the plasma membrane, supporting the endosomal mode of exosome biogenesis. Electron microscopy [40] and genetic studies [41, 42] have provided considerable evidence for the endosomal mode of exosome biogenesis. Since no specific markers of EV subtypes have been defined and ultracentrifugation is incapable of distinguishing exosomes from MVs, EVs which have only detected surface markers or sizes can not be considered as exosomes [43]. Therefore, we used the general term EVs instead of exosomes in this review unless authors have performed studies to determine that the EVs were of endosomal origin.

The biogenesis of EVs determines their characteristics including the heterogeneity in size, content, biological marker, source and function. Combined asymmetric-flow field-flow fractionation with real-time monitors revealed that exosomes may be classified into two distinct subsets: large exosomes (90–120 nm in diameter) and small exosomes (60–80 nm in diameter) [44, 45]. Meanwhile, EVs can be secreted by multiple cells types [46, 47], including macrophages [48], nervous cells, endothelial and epithelial cells [49], and mesenchymal stem cells [50, 51], which lead to the inclusion of different kinds of proteins, lipids and nucleic acids, and the expression of different detectable markers (CD81, CD82, CD37 and CD63) on EVs [52]. EVs with different labels can transfer contents to different recipient cells and tissues. Therefore, the heterogeneity of EVs contributed to the diversity and complexity of their function.

## The functional role of EV ncRNAs in gynecological malignancy

Ratajczak et al. [53] first reported that mRNAs are selectively enriched in MVs derived from murine embryonic stem cells versus parental cells and that these mRNAs are further delivered to target cells to upregulate the expression of protein markers. Later, Valadi et al. reported that EVs derived from human and murine cell lines contain both mRNAs and small RNAs and that EV mRNAs transferred to other cells can also be translated to proteins in new cells, suggesting that EV mRNAs still function after transfer to the recipient cells [54]. Accumulating evidence has shown that EVs contain multiple types of ncRNAs, mainly miRNAs, lncRNAs and circRNAs. These EV ncRNAs, delivered to recipient cells by EVs, exert their functions and affect the cell phenotype, playing a significant role in various biological functions, particularly in cancer development.

**Fig. 2:**
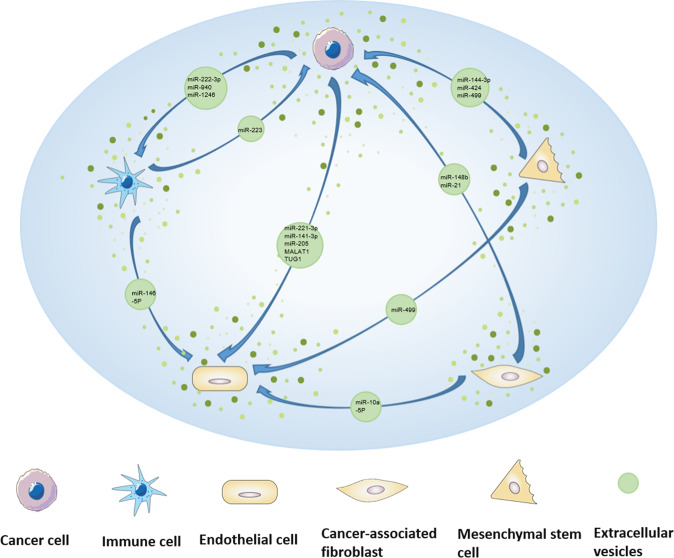
EV ncRNAs mediate the communication between cancer cells and stromal cells in TME of gynecological malignancies. ncRNAs derived from cancer or stromal cells are packaged in EVs are shuttled among cancer cells and different stromal cells, remodeling TME and contributing to the cancer development and metastasis.

EV ncRNAs derived from cancer or stromal cells serve as mediators among these cells, constitute critical component of tumor microenvironment (TME). EV ncRNAs from cancer cells can be transported not only to cancer cells, but also to other stromal cells, such as endothelial cells and immune cells. For example, EVs from CC and OC cells transported miR-221-3p [55–57], miR-141-3p [58], TUG1 [59] or MALAT1 [60] to human lymphatic endothelial cells (HLECs), microvascular endothelial cells (MVECs), human umbilical vein endothelial cells (HUVEC) or other endothelial cells, thus promoting endothelial cells proliferation, migration and tube formation, which facilitates to angiogenesis and cancer metastasis. And packaged in EVs, miR-21 [61] in EC and miR-1246 [62], miR-940 [63] and miR-222-3p [64] in OC can be transported to THP-1 or macrophages, which stimulated M2 phenotype polarization of macrophage, thus remodeling the tumor immune microenvironment and promoting cancer development. Meanwhile, miR-1246 was abundantly expressed in EVs derived from paclitaxel resistance-OC cells and miR-1246 inhibitor in combination with paclitaxel reduced tumor growth in mouse model, suggesting EV miR-1246 shuttling between cancer cells contributed to chemoresistance of OC. Besides, cancer-associated fibroblasts (CAFs)-secreted EV ncRNAs can also be delivered to cancer cells and endothelial cells, promoting cancer cell proliferation, chemoresistance and angiogenesis, such as EV miR-98-5p [65], EV miR-21 [66] and EV miR-10a-5p [67]. In contrast, EV miR-146-5p from tumor-associated macrophage (TAM) and EV miR-424 from mesenchymal stem cell (MSC) can be transferred to HUVEC to inhibit angiogenesis [68, 69]. In addition, human bone marrow mesenchymal stem cells (hBMSCs) and MSC can also deliver EV ncRNAs to cancer cells to restrain the proliferation, migration, and invasion of cancer cells [69, 70]. However, normal cells can also secret EV ncRNAs and play a role in cancer inhibition. Human ovarian surface epithelial cells-secreted EV miR-124 can be transported to CAFs to reverse the transdifferentiation of normal fibroblasts (NFs) to CAFs and affect ECM remodeling [71]. All above findings demonstrated that EV ncRNAs mediated intercellular communication in the TME (Fig. 2), between cancer cells and stromal cells, which further remodeled TME and contributed to the cancer development and metastasis.

**Fig. 3:**
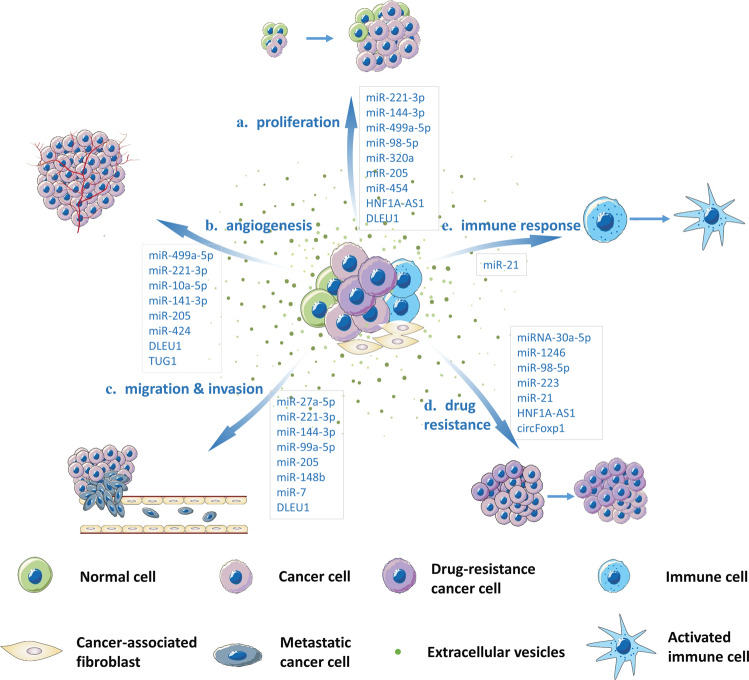
A schematic representation of EV ncRNAs in regulation of gynecological malignancies. EV ncRNAs released by different cells regulate tumorigenesis and progression by promoting cancer cell proliferation, migration, invasion drug resistance, angiogenesis and immune response.

In summary, EV ncRNAs mostly regulate tumorigenesis and progression in following ways (Fig. 3): they (a) accelerate cancer progression by promoting cancer cell proliferation, (b) facilitate angiogenesis by altering the phenotype of endothelial cells, (c) promote metastasis and invasion by mediating EMT and transferring EV ncRNAs to distant tissues, (d) spread chemoresistance among heterogeneous populations of cancer cells, and (e) regulate the immune response. It has been reported that a large number of EV ncRNAs, listed in Table 1, function in the above ways in gynecological malignancies. Next, we summarized the functional roles of EV ncRNAs in CC, OC and EC.

**Table 1:** Functions of EV ncRNAs in gynecological malignancies.

Cancer type	Origin	ncRNAs	Function	Pathway/Target	Reference
CC	CC cells	miR-221-3p	Lymphangiogenesis and lymphatic metastasis	VASH1	Zhou et al. [55]
CC	CC cells	miR-221-3p	Proliferation, invasion, migration and angiogenesis	MAPK10	Zhang et al. [56]
CC	CC cells	miR-221-3p	Angiogenesis	THBS2	Wu et al. [57]
CC	Human bone marrow mesenchymal stem cells	miR-144-3p	Proliferation, invasion and migration	CEP55	Meng et al. [70]
CC	CC cells	miR-1468-5p	Immunosuppressive	JAK2/STAT3	Zhou et al. [116]
CC	Cancer-associated fibroblast	miR-10a-5p	Angiogenesis	TBX5 mediated Hh signaling	Zhang et al. [67]
CC	DDP‑resistant Hela cells	HNF1A‑AS1	Proliferation, drug resistance, apoptosis	microRNA-34b/TUFT1	Luo et al. [83]
CC	CC cells	TUG1	Angiogenesis	angiogenesis-related genes	Lei et al. [59]
OC	Hypoxic macrophages	miR-223	Drug resistance	PTEN-PI3K/AKT	Zhu et al. [117]
OC	Human ovarian surface epithelial cells	miR‐124	Extracellular matrix remodeling	SPHK1	Zhang et al. [71]
OC	OC cells	miR-99a-5p	Invasion	HPMCs	Yoshimura et al. [96]
OC	SKOV3	miR-205	Proliferation, migration, invasion, apoptosis and EMT progression	VEGFA	Wang et al. [94]
OC	OC cells or serum	miR-205	Angiogenesis and metastasis	PTEN-AKT	He et al. [95]
OC	MDA-MB-231	miR-454	OC cell growth	PRRT2	Wang et al. [98]
OC	Human adipose mesenchymal stem cells	miRNAs	Apoptosis	pro-apoptotic signaling molecules	Reza et al. [118]
OC	SKOV3	miR-141-3p	Angiogenesis	JAK/STAT3	Masoumi-Dehghi et al. [58]
OC	IOSE-80	miR-34b	Proliferation and EMT	Notch2	Lu et al. [99]
OC	SKOV3/DDP cells	miR-30a-5p	Drug resistance	SOX9	Liu et al. [119]
OC	OC cells	miR-1246	Drug resistance	Cav1/p-gp/M2-type macrophage axis	Kanlikilicer et al. [62]
OC	TWEAK-stimulated macrophages	miR-7	Metastasis	EGFR/AKT/ERK1/2	Hu et al. [101]
OC	Cancer-associated fibroblast cells	miR-98-5p	Proliferation, cell cycle and drug resistance	CDKN1A	Guo et al. [65]
OC	Cancer-associated adipocytes and fibroblasts cells	miR-21	Apoptosis and drug resistance	APAF1	Au Yeung et al. [66]
OC	Mesenchymal stem cells	miR-424	Tumorigenesis and angiogenesis	MYB	Li et al. [69]
OC	Serum	circFoxp1	Drug resistance	miR-22 and miR-150-3p/ CEBPG and FMNL3	Luo et al. [97]
EC	Cancer-associated fibroblasts cells	miR-320a	Proliferation	HIF1α/VEGFA	Zhang et al. [108]
EC	Hypoxic KEL cells	miR-21	M2 polarization	IL-10 and CD206	Xiao et al. [61]
EC	Cancer-associated fibroblasts cells	miR‐148b	Invasion and metastasis	DNMT1	Li et al. [109]
EC	Serum	miR-27a-5p	Migration and invasion	SMAD4	Che et al. [120]
EC	Mesenchymal stem cells	miR-499a-5p	Proliferation, tumor growth and angiogenesis	VAV3	Jing et al. [121]
EC	EC cells	DLEU1	Proliferation, migration, invasion	microRNA/E2F3	Jia et al. [110]

## EV ncRNAs in CC

CC continues to be a common gynecological malignancy. In 2018, there were 569,847 new cases and 311,365 deaths worldwide, making it the fourth most harmful malignancy threatening women’s health and lives [72]. Persistent infection with high-risk human papilloma virus (HPV) is the main risk factor, and HPV infection can be detected in nearly 99% of CC tissues; HPV 16 and 18 account for ~70% of HPV infections [73]. At present, the popularization of tertiary prevention makes CC controllable, but the prognosis of recurrent and metastatic CC is poor.

HPV infection affects the number and content of EVs and leads to the selective packaging of several miRNAs and lncRNAs into EVs[74]; the viral E6/E7 oncogene plays an important role in this process [75, 76]. EVs were found to be enriched in cervical-vaginal lavage specimens, and it was reported that 45 miRNAs were significantly upregulated and 55 miRNAs were significantly downregulated in EVs from cervical-vaginal lavage specimens from HPV 16-positive patients versus those from healthy volunteers [77]. In particular, EV miR-21 and miR-146a were markedly elevated in CC patients [78]. The lncRNAs HOTAIR, MALAT1 and MEG3 were also enriched in EVs derived from cervical-vaginal lavages and significantly upregulated in CC patients compared with cancer-free volunteers [79]. In plasma specimens, Zheng et al. reported that EV miR-30d-5p and let-7d-3p distinguished not only CC patients from normal patients but also cervical intraepithelial neoplasia (CIN) II+ group patients from CIN I− group patients [80]. In addition, the expression of lncRNA-EXOC7 was significantly upregulated in EVs from the serum and correlated with the International Federation of Gynecology and Obstetrics (FIGO) stage [81]. These ncRNAs may serve as biomarkers for monitoring cancer occurrence and development. Compared to traditional solid biopsies, liquid biopsies have gained increasing attention as they can be performed easily and noninvasively [82].

In CC, EVs transfer ncRNAs to different recipient cells to play different roles in cancer development. For example, Zhou et al. reported that cervical squamous cell carcinoma (CSCC) cells secret EVs to accelerate cancer progression and metastasis by transferring miR-221-3p into different epithelial cells [55, 57]. EV miR-221-3p was delivered into human lymphatic endothelial cells (HLECs) to promote lymphangiogenesis and lymphatic metastasis by targeting vasohibin-1 (VASH1) [55], and delivered into human umbilical vein endothelial cells (HUVECs) to promote angiogenesis by downregulating THBS2 [57]. Zhang et al. reported that EV miR-221-3p can also be transferred into microvascular endothelial cells (MVECs) to promote cell proliferation, invasion and migration by upregulating MAPK10 [56]. It was also reported that TUG1 is overexpressed in both CC cells and their secreted EVs [59]. EV TUG1 transferred to HUVEC promotes angiogenesis by regulating certain key angiogenesis-related genes. EV TUG1 also promotes HUVEC proliferation by regulating apoptosis-related proteins. Furthermore, EV ncRNAs participate in cancer drug resistance. Luo et al. reported that EVs secreted from cisplatin (DDP)-resistant HeLa cells transfer HNF1A‑AS1 to DDP‑sensitive HeLa cells. EV HNF1A‑AS1 sponges miR‑34b to upregulate TUFT1 expression, promoting the proliferation and drug resistance of cancer cells [83]. These studies not only showed the important role that EV ncRNAs play in CC development but also provided promising targets for the diagnosis and treatment of CC.

Finally, EVs can also be employed as shuttles to transfer functional moleculars to specified cells or tissues. Federico et al. delivered an anti-HPV16-E7 single-chain variable fragment (scFv) into cells to inhibit the proliferation of HPV16-E7-expressing cells by engineered EVs [84]. Unlike liposomes, which may induce undesired immune responses leading to low transport efficiency, endogenous EVs secreted by various cells are considered a natural ideal delivery system [85].

## EV ncRNAs in OC

OC is one of the three major gynecological malignancies and has the highest mortality rate [86]. Owing to the lack of clinical symptoms in the early stage, more than 80% of OC patients are diagnosed at an advanced stage, always accompanied by metastasis to the peritoneal cavity or upper abdominal organs, leading the 5-year overall survival (OS) rate for stage III and IV disease is less than 30% [87]. Thus, there is an urgent need to develop biomarkers for the early diagnosis of OC.

A multitude of studies have reported that EV ncRNAs are differentially expressed in serum, plasma and ascites samples between normal and OC patients. It has been reported that the expression of serum EV miR-484 in OC patients is significantly decreased, which is not only closely related to cancer progression, but is also an independent predictor for OS and progression-free survival (PFS) of OC patients [88]. In contrast, the high expression of EV aHIF and MALAT1 in the serum of OC patients may predict poor OS [60, 89]. However, serum EV miR-375, miR-1307 and miR-34a may be used as diagnostic biomarkers due to their abnormal expression in patients with advanced stage disease or with lymph node metastasis [90, 91]. In addition, EV ncRNAs in plasma, ascites, or cancer cells may also be used as biomarkers. miR-21, miR-100, miR-200b, and miR-320 were all upregulated in the plasma of OC patients, and miR-200b was closely related to patient OS [92]. Differential expression of EV miRNAs was also detected in ascites from OC patients, among which miR-149-3p and miR-222-5p were significantly correlated with 5-year survival and OS [93]. The aforementioned ncRNAs are enriched in EVs and can be easily collected and detected, so they may serve as potential biomarkers to improve the efficiency of diagnosis and disease for OC.

EV ncRNAs also regulate OC development by acting as oncogenes or tumor suppressors. On the one hand, it has been reported that EV miR-205 and miR-141-3p derived from OC cells promote cell proliferation, migration, invasion and angiogenesis [58, 94, 95]. In particular, EV miR-99a-5p derived from epithelial ovarian cancer (EOC) cells can be transferred to neighboring human peritoneal mesothelial cells and upregulate the expression of fibronectin and vitronectin, promoting OC cell invasion [96]. Additionally, multiple types of cells secrete EV ncRNAs to promote sensitive cancer cell chemoresistance. Yeung et al. reported that EV miR-21 derived from cancer-associated adipocytes (CAAs) and fibroblasts (CAFs) confers cancer cell chemoresistance by binding to APAF1 [66]. Luo et al. also reported that circulating EV circFoxp1 confers EOC cell chemoresistance [97]. In addition, MDA-MB-231 cells transfer EV microRNA-454 to promote the stemness of cancer stem cells in OC [98]. Therefore, these EV ncRNAs promote OC progression by regulating the proliferation, migration, invasion, and chemoresistance of cancer cells.

On the other hand, EV ncRNAs, such as EV miR-34b, miR-124 and miR-6126, function as tumor suppressors in OC progression [71, 99, 100]. For example, miR‐124 transfer from human ovarian surface epithelial cells to CAFs leads to the attenuation of cell motility and invasion. Specifically, miR‐124 binds to the 3’ UTR of SPHK1 mRNA to inhibit SPHK1 expression, which promotes the transition of normal fibroblasts to CAFs, thereby contributing to extracellular matrix (ECM) remodeling [71]. Another example is EV miRNA-7 derived from tumor necrosis factor (TNF)-like weak inducer of apoptosis (TWEAK)-stimulated macrophages, which inhibits cancer metastasis after transfer to EOC cells [101]. The overexpression of these miRNAs inhibits cancer progression, suggesting they may provide new therapeutic approach to cancer treatment.

## EV ncRNAs in EC

Both the incidence and mortality rates of EC, the most common gynecological malignancy in developed countries, are increasing [3, 102]. It was predicted that by 2030, the EC incidence rate is expected to reach over 42.13 cases per 100,000 women [103]. Based on the clinical features, grade, hormone receptor expression, histology and other related factors, EC is divided into two classes: type I and type II. Unlike type I, which has a good prognosis, type II EC is usually diagnosed at an advanced stage, accompanied by distant metastasis, which indicates a poor prognosis [104].

Although studies on EV ncRNAs in EC are limited, the existing data provide a starting point for fully characterizing these components in EC. Similar to CC and OC, certain ncRNAs are enriched in EVs from EC patients. It has been reported that circRNAs are differentially expressed in serum EVs between EC patients and normal controls [105]. The urine-derived EVs in EC patients revealed that miRNAs are also be selectively packaged into EVs and that miR-200c-3p was significantly enriched in these EVs [106]. In addition, 114 miRNAs were differentially expressed in EVs derived from peritoneal lavage samples from EC patients compared with control patients, and among these miRNAs 18 were upregulated and 96 were downregulated. Importantly, 8 significantly downregulated miRNAs were found to have predictive value [107]. These EV ncRNAs provide a novel avenue for improving the clinical diagnosis and management of cancer.

It was reported that cancer-associated fibroblast (CAFs)-secreted EV miRNAs can suppress cancer progression. Zhang et al. reported that EV miR-320a secreted by CAFs inhibits cancer cell proliferation via HIF1α/VEGFA axis [108]. Similarly, EV miR‐148b derived from CAFs suppresses EC metastasis, by directly binding to DNMT1 [109]. However, downregulated miR-320a and miR‐148b in CAFs and CAFs‐derived exosomes induced EMT to promote endometrial EC progression. In contrast, EV ncRNAs may also accelerate EC progression. The overexpression of miR-381-3p in tumor tissues was found to reduce *E2F3* expression to inhibit the proliferation, migration, and invasion of EC cells. However, EV DLEU1 accelerate EC progression by negatively regulating the miR-381-3p/E2F3 axis [110]. Furthermore, EV miR-21, which was secreted from EC KEL cells at a higher level under hypoxic conditions than under normoxic conditions, was transferred into THP-1 cells to promote M2-like macrophage polarization, suggesting that EV ncRNAs participate in the cancer immune microenvironment [61]. These results suggest that EV ncRNAs also play a significant role in EC progression. As our understanding of EV ncRNAs increases, new biomarkers or therapeutic approaches to improve EC management will be revealed.

## Conclusion and future perspectives

Similar to their application in other cancers, the clinical applications of EV ncRNAs in gynecological malignancies are mainly focused on two aspects. Increasing evidence has shown that EV ncRNAs have the potential to serve as biomarkers for cancer diagnosis as well as for the prediction and monitoring of therapeutic effects (Table 2). The membrane structures of EVs provide a shell to protect ncRNAs from degradation by endogenous RNase. EVs can be secreted into serum, urine, saliva, ascites, pleural fluid and so on. These biological fluids are easy to sample, making EV ncRNAs ideal biomarkers. Importantly, EVs can be secreted by almost all kinds of cells, indicating that they can reflect the pathological and physiological conditions of donor cells. Therefore, EV ncRNA detection through liquid biopsy provides a novel method for cancer diagnosis and monitoring.

**Table 2:** Potential biomarker EV ncRNAs in gynecological malignancies.

Cancer type	Source	ncRNAs	Biomarker	qPCR verification	Reference
CC	Plasma	Let-7d-3p miR-30d-5p	Diagnosis	YES	Zheng et al. [80]
CC	Plasma	miR-125a-5p	Diagnosis	YES	Lv et al. [122]
CC	Serum	lncRNA-EXOC7	Diagnosis, treatment effect and recurrence	YES	Guo et al. [81]
OC	Serum	miR-484	Prognosis	YES	Zhang et al. [88]
OC	Serum	miR-145, miR-200c	Diagnosis	YES	Kim et al. [123]
OC	Serum	aHIF	Prognosis	YES	Tang et al. [89]
OC	Serum	miR-1307 miR-375	Diagnosis	YES	Su et al. [90]
OC	Serum	MALAT1	Prognosis	YES	Qiu et al. [60]
OC	Serum	miR-373, miR-200a, miR-200b, miR-200c	Diagnosis, prognosis	YES	Meng et al. [124]
OC	Serum	miR-34a	Prognosis	YES	Maeda et al. [91]
OC	Serum	miR-1290	Prognosis	YES	Kobayashi et al. [125]
OC	Plasma	miR‑93‑5p miR-122-5p miR‑99b‑5p	N.D	YES	Zhang et al. [126]
OC	Plasma	miR-23a, miR-92a miR-21, miR-100, miR-200b miR-16	Diagnosis, prognosis	No	Pan et al. [92]
OC	Ascites	miR-149-3p, miR-222-5p	Prognosis	YES	Li et al. [93]
OC	OC cells	let-7, miR-200 miRNA family	Invasiveness	YES	Kobayashi et al. [127]
EC	Peritoneal lavage	miR-383-5p, miR-10b-5p, miR-34c-3p, miR-449b-5p, miR-34c-5p, miR-200b-3p, miR-2110, and miR-34b-3p	N.D	NO	Roman-Canal et al. [107]
EC	Serum	209 upregulated and 66 downregulated circRNAs	N.D	Confirmed (hsa circ 0109046 and hsa circ 0002577)	Xu et al. [105]
EC	Urine	miR-200c-3p	N.D	YES	Srivastava et al. 2018 [106]

*N.D* not determined.

Due to the natural characteristics of EVs and the development of engineering technology, EVs have been suggested to have potential applications in cancer treatment. EV ncRNAs have a vital role in cancer progression and chemoresistance, which may be restrained by inhibiting the release and uptake of cancer-related EVs. Because certain membrane proteins are overexpressed in cancer-related EVs, EVs can also be employed as shuttles to transfer functional moleculars to specified cells or tissues. It has been reported that the nonapeptide LXY30 can reduce EV uptake into OC cells by binding to the overexpressed membrane protein α3β1 integrin [111]. In addition, unlike liposomes, which may induce undesired immune responses leading to low transport efficiency, endogenous EVs secreted by various cells are considered a natural ideal drug delivery system [85]. Aqil et al. employed milk EVs to deliver plant bioactive berry Anthos, which enhanced the drug’s antiproliferative activity against OC cells by enhancing its oral bioavailability [112]. Zhang et al. reported that umbilical cord-derived macrophage EVs loaded with cisplatin increased their cytotoxicity in both drug-resistant and drug-sensitive OC cells, significantly improving the efficacy of cisplatin [113]. This emerging therapeutic approach can improve drug loading, enhance endocytosis, lower toxicity and protect contents from degradation, making EVs an ideal drug delivery system. Moreover, investigating the mechanisms by which EV ncRNAs confer chemoresistance could improve our knowledge about chemoresistance, providing more treatment opinions. Furthermore, EVs may be developed as vaccines to stimulate anticancer immunity. EV-based vaccines may stimulate both adaptive and innate immunity. The group of Maurizio Federico engineered endogenous EVs loaded with HPV E7 protein, which induces a protective CD8+ T cell immune response [114]. They also produced immunogenic EVs in vivo by injection of Nef^mut^/E7 DNA to elicit an E7-specific cytotoxic T lymphocyte (CTL) immune response [115]. Above all, these results represent emerging novel applications of EVs in cancer therapy.

Taken together, these findings indicate that EV-delivered ncRNAs extensively participate in the development of gynecological malignancies. Many studies have attempted to elucidate the mechanism by which EV ncRNAs are produced and their biological roles in the occurrence and progression of gynecological malignancies. Numerous studies have also striven to employ these EV ncRNAs to diagnose or monitor cancer progression. Some studies have even applied EVs to treat gynecological malignancies or elicit cancer-associated immune responses. Although these applications are under development and exploration, they bring new ideas and hope for improving the diagnosis and treatment of gynecological malignancies.

## Data Availability

Data sharing is not applicable to this article as no datasets were generated or analyzed during the current study.
